# First Use of AXL Targeting in Metastatic, Refractory, Adenoid Cystic Carcinoma: A Case Report

**DOI:** 10.1200/PO.23.00633

**Published:** 2024-04-05

**Authors:** Camilla O. Hoff, Eduardo Andreazza Dal Lago, Juliana Mota Siqueira, Luana G. de Sousa, Adel K. El-Naggar, Jordi Rodon Ahnert, Renata Ferrarotto

**Affiliations:** ^1^ Department of Thoracic Head and Neck Medical Oncology, The University of Texas MD Anderson Cancer Center, Houston, TX; ^2^ Department of Thoracic Imaging, The University of Texas MD Anderson Cancer Center, Houston, TX; ^3^ Department of Pathology, The University of Texas MD Anderson Cancer Center, Houston, TX; ^4^ Department of Investigational Cancer Therapeutics, The University of Texas MD Anderson Cancer Center, Houston, TX

## Abstract

First use of AXL-targeting in adenoid cystic carcinoma (ACC); with positive results, ACC now included in AXL studies.

## Introduction

Adenoid cystic carcinoma (ACC) is a secretory gland cancer, most frequently from the upper aerodigestive tract.^1,2^ Despite aggressive curative-intent primary therapy, approximately 50% develop distant metastasis.^3-6^ Treatment of metastatic ACC is challenging, with no US Food and Drug Administration (FDA)–approved agents.^3,7,8^ The development of effective and tolerable therapies is an urgent need.

ACC is highly heterogenous and can be classified into two subtypes.^9-11^ ACC-I is aggressive, with a median overall survival (mOS) of 3.4 years and enrichment for *NOTCH1*-activating mutations and solid histology.^11,12^ ACC-II is more indolent, with a mOS of 23.2 years, *TP63* overexpression, predominantly nonsolid (tubular/cribriform) histology, and tendency for lung metastasis.^11^ ACC-II is more frequent, representing approximately 70% of ACC. Proteogenomic characterization of the two subtypes led to discovery of potential subtype-specific therapeutic targets. In ACC-II, *AXL* gene expression and protein expression are significantly increased, providing a rationale for AXL-targeted therapy.^11,13,14^

Mecbotamab vedotin (BA3011) is an AXL-targeting, conditionally active biologic antibody-drug conjugate that delivers the cytotoxic agent monomethyl auristatin E (MMAE).^15,16^ BA3011 is designed to bind to AXL under tumor-specific conditions of accelerated glycolytic metabolism.^15-17^ Considering that AXL is expressed physiologically in many tissues, CAB-ADCs have the potential advantage of increased tumor specificity.^15-17^

Here, to our knowledge, we describe the first patient with ACC treated with AXL-targeted therapy (BA3011) as part of a phase I study (ClinicalTrials.gov identifier: NCT03425279), after identification of her ACC-II disease type.

## Case Report

In April 2018, a 28-year-old, never-smoker woman with no comorbidities developed dyspnea on mild-to-moderate exertion and an anterior midline neck mass. Neck ultrasound detected a 3.5-cm solid nodule invading the thyroid isthmus, with initial pathology favoring tracheal ACC (Data Supplement). Chest computed tomography (CT-C) showed multiple small (≤5 mm) bilateral lung nodules, indeterminate but concerning for metastasis. The patient underwent a total thyroidectomy with resection of the first three tracheal rings and postoperative radiotherapy (60 Gy), completed in September 2018. Surgical specimen pathology confirmed ACC with cribriform histology, 2.8 cm, close surgical margins and perineural invasion, negative for PD-L1 (clone 22C3, Dako, Carpinteria, CA), and pathologic staging pT4aN0 (American Joint Committee on Cancer staging, eighth edition).

**FIG 1.**
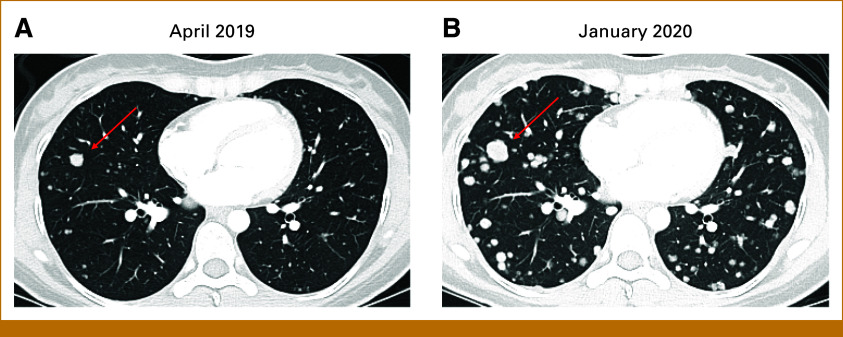
Chest CT shows progression of ACC lung metastases and the development of pleural lesions while on active surveillance over a period of 9 months. (A) April 2019: multiple bilateral nodules, with the largest measuring 10.5 mm (arrow). (B) January 2020: progressive growth of existing lesions and appearance of multiple new lesions and nodular pleural thickening (not shown). The previous largest lesion grew from 10.5 mm to 16.7 mm (arrow), representing an increase of 59%. ACC, adenoid cystic carcinoma; CT, computed tomography.

Serial CT-C during the following months demonstrated growth of existing lung nodules and appearance of new lesions. In April 2019, imaging showed innumerable lung nodules, largest 1.1 cm (Fig 1A). Biopsy confirmed metastatic ACC, PD-L1–positive in 1% of tumor cells, with no copy number variations or somatic mutations on MDACC Genomic Assay (Data Supplement), including no mutations in *NOTCH* genes. Patient remained on close surveillance and asymptomatic.

In January 2020, CT-C revealed pleural disease and a continuous increase in lung nodules (Fig 1B). The patient-physician decision was to begin systemic therapy in a phase II trial of VEGFR inhibitor (VEGFRi) axitinib plus PD-L1 inhibitor avelumab.^18^ Best overall response (BOR) was stable disease (SD) with +14% target lesion (TL) growth, with progression (PD) after 7 months. In October 2020, she entered a phase I PRMT5 inhibitor (PRMT5i) trial. BOR was SD with maximum shrinkage –16% from baseline. Although she remained with SD, lung and pleural lesions continued to grow accompanying progressive dyspnea, with the patient making the psychologically difficult decision to request disability leave from work. Given progression clinically and in multiple non-TL, PRMT5i was interrupted in November 2021.

**FIG 2.**
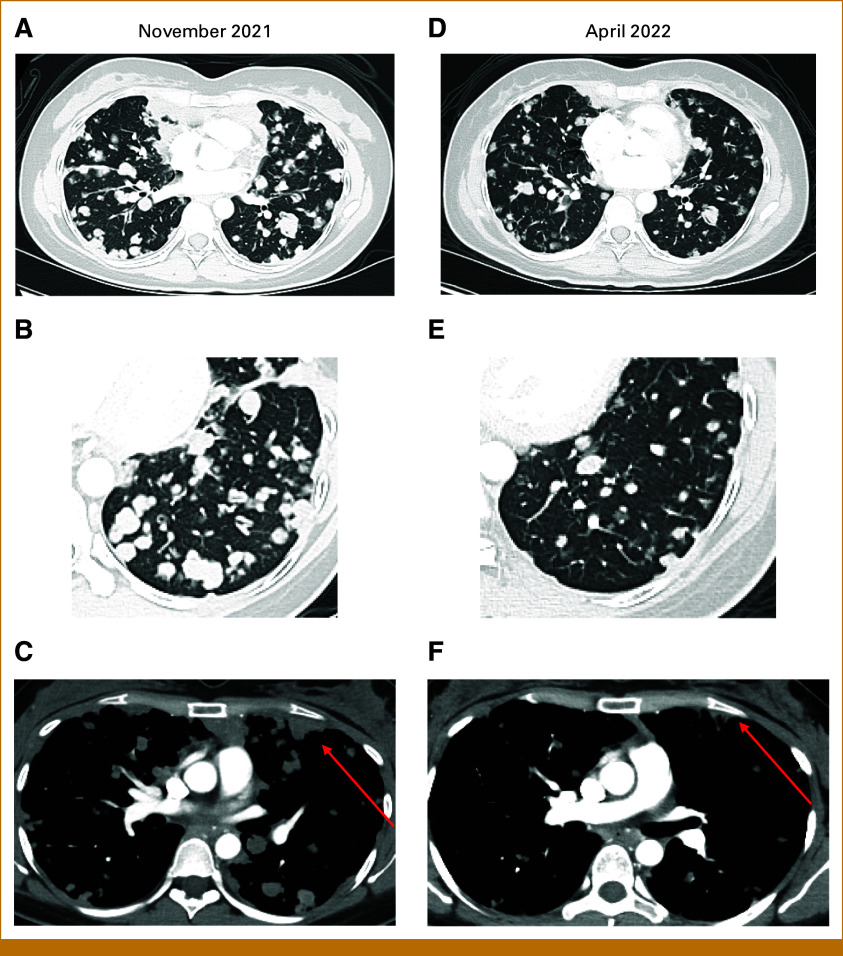
Chest CT showing response to treatment with AXL-CAB-ADC. Axial images with lung window show a reduction in the size and number of parenchymal metastatic nodules (A and B) from the baseline examination (November 2021) (D and E) to the on-treatment examination (April 2022). In addition, (C) chest CT with contrast (mediastinal window) reveals metastatic pleural disease (arrow) characterized by nodular pleural thickening in the baseline examination (November 2021). (F) Post-treatment examination (April 2022) reveals a significant decrease in the pleural lesions (arrow). CT, computed tomography.

At this point, the patient was highly symptomatic, with dyspnea at rest, requiring continuous oxygen at 2-4 L. CT-C revealed innumerable bilateral parenchymal and pleural metastases, largest measuring 3.9 cm (Figs 2A-2C).

**FIG 3.**
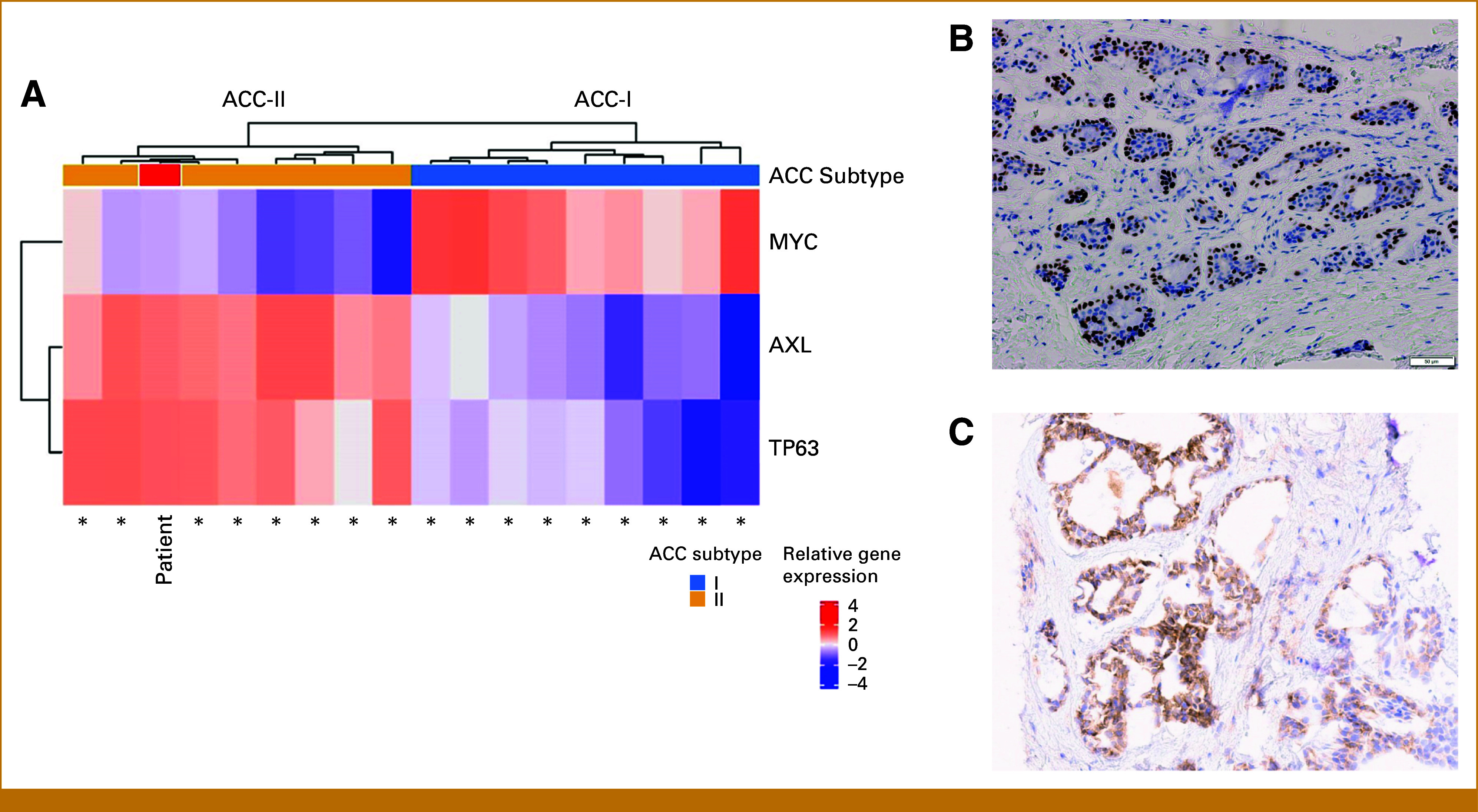
Gene and protein expression profiles reveal that patient has ACC-II subtype, AXL-high. (A) TP63, MYC, and AXL gene expression in an 18-patient ACC cohort, including our index patient (red). TP63 and MYC relative gene expression is sufficient to distinguish between ACC-I and II subtypes, as ACC-II has relatively higher expression of TP63 than MYC, seen in our patient. AXL gene expression is higher in ACC-II. RNA sequencing performed with HTG Molecular Diagnostics, Inc, Tucson, AZ. (B) P63 IHC Expression. Nuclear immunostaining of P63 (4A4; CM163C; Biocare, Pacheco, CA) in approximately 60% of tumor cells, classified as P63 positive. (C) AXL IHC expression. IHC analysis of AXL expression (7E10; ab130218; Abcam, Cambridge, United Kingdom) revealed strong and diffuse staining in over 70% of tumor cells and negative expression in stroma cells. ACC, adenoid cystic carcinoma; IHC, immunohistochemistry.

To evaluate potential new clinical trials, we investigated patient ACC subtype. Given cribriform histology and metastases confined to lungs, we hypothesized that she had ACC-II.^11^ ACC can be subtyped using *MYC* and *TP63* gene expression. ACC-II presents relatively higher expression of *TP63* than *MYC*,^11^ seen in this patient (Fig 3A). In addition, P63 immunohistochemistry (IHC), a robust surrogate marker for ACC subtype,^11^ was positive in primary tumor and lung metastasis (Fig 3B), further indicating ACC-II.

Given association of ACC-II with high AXL expression,^11^ the sponsor of the AXL-ADC BA3011 trial allowed us to screen her for their trial, which required high AXL expression. IHC was AXL-high (Fig 3C), and she enrolled in the trial dose expansion.

In December 2021, to our knowledge, our patient became the first patient with ACC treated with BA3011, receiving a dose of 1.8 mg/kg administered intravenously once every 2 weeks. Within weeks, she experienced significant clinical improvement: improved lung expansibility; return of hiccups and functional sneezes, previously lost because of pleural disease; and increase in energy allowing treadmill walking with oxygen. Consistently, first images in January 2022 and March 2022 revealed a –3% and –10% TL reduction from baseline. Dyspnea continuously improved, with patient weaning off oxygen, exercising at a healthy pace, and even choreographing a dance number for a family wedding. In April 2022, CT-C revealed further reduction in TL to –25% from baseline and disappearance of some lung nodules (Figs 2D-2F). Although SD per RECIST, the radiologic improvement in disease burden and remarkable clinical improvement were consistent with a significant response to therapy, persistent throughout BA3011 use. RECIST remained SD throughout 2022, and she remained off oxygen. Throughout treatment, antidrug antibody (ADA) titers were assessed, remaining negative until before cycle 10, when they became positive and continuously increased, reaching a peak precycle 12 in November 2022.

**FIG 4.**
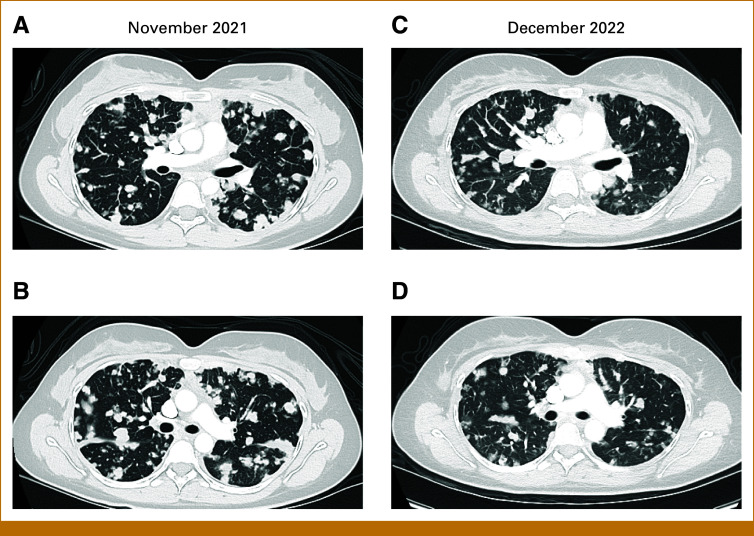
Chest CT showing pulmonary tumor burden (A and B) before treatment with AXL-CAB-ADC ( November 2021) compared with (C and D) post-treatment (December 2022). Despite progression documented as over 20% growth from nadir, imaging reveals overall improvement in ACC pulmonary disease. ACC, adenoid cystic carcinoma; CT, computed tomography.

During December 2022, the patient reported increased dyspnea with moderate exertion. Radiologic progression from nadir occurred at the end of December 2022, and patient came off trial. At RECIST PD, her disease burden and functional status were much improved as compared with before AXL-ADC therapy (Fig 4).

AXL-ADC adverse events, evaluated by Common Terminology Criteria for Adverse Events, were mostly intermittent grade 1 (G1), with isolated grade 2 (G2). During initial cycles, the patient reported G2 fatigue, nausea, and vomiting (N/V). Both fatigue and N/V stabilized as G1, with the latter well-managed pharmacologically. Laboratorial abnormalities included G1 anemia, elevated liver enzymes, and proteinuria, with one episode of G2 anemia. After 3 months on therapy, the patient acquired premature ovarian insufficiency with intermittent nocturnal hot flashes. After 7 months, she developed bilateral lower extremity neuropathy. Although initially intermittent, the neuropathy became constant and reached G2 in September 2022, with dose reduced to 1.5 mg/kg administered intravenously once every 2 weeks, after which neuropathy stabilized as G1.

## Ethics Approval

This study was conducted in accordance with the Declaration of Helsinki. Samples were obtained under The University of Texas MD Anderson Cancer Center (MDACC) Institutional Review Board (IRB)–approved informed consent for molecular and clinicopathologic analyses with approved IRB protocols (PA-17-0865 and 2019-0107).

## Statement of Patient Consent

The patient gave informed consent for analysis of tumor specimens and publication of this report.

## Discussion

Here, we present a young patient with ACC with predominantly cribriform histology and lung metastasis, a frequent ACC presentation highly suggestive of ACC-II subtype. RNA sequencing and IHC confirmed ACC-II classification, with increased TP63 gene and protein expression. Despite classification as ACC-II, our patient experienced relatively rapid progression of pulmonary metastatic disease, a more aggressive presentation within the ACC-II spectrum. Beyond prognostic significance, ACC subtype has potential therapeutic implications.^19^ ACC-II has significantly upregulated AXL^11^ when compared with ACC-I.^11^ Our ACC-II patient tumor was confirmed AXL-high (Fig 3).

AXL is overexpressed in many cancers^20^ and promotes tumor proliferation, survival, and epithelial-to-mesenchymal transition (EMT). EMT participates in metastasis development^21,22^ and formation of cancer stem cells, associated with disease recurrence and chemotherapy and immunotherapy resistance.^23-26^ In ACC, EMT has been linked to perineural invasion,^27^ metastasis,^28^ and radioresistance.^29^ While AXL overexpression is associated with the better prognosis ACC-II,^11^ this subtype has frequent pulmonary metastasis, which may be related to EMT.^30,31^ Given AXL overexpression in the largest ACC subtype group, there is significant biologic rationale to support investigation of AXL-targeted therapy in ACC. Accordingly, AXL targeting is cytotoxic in AXL-high preclinical ACC models.^13,14^

Currently, there is no FDA-approved systemic therapy for ACC.^19,32,33^ Despite well-documented heterogeneity, all ACCs are treated similarly, with VEGFRi and chemotherapy being the only options outside of clinical trials. On trial, our patient received VEGFRi plus PD-L1 inhibitor as first-line treatment and PRMT5i as second-line treatment. For both, BOR was SD although lung and/or pleural lesions continued to grow below RECIST progression threshold and accompanied clinical deterioration.

To our knowledge, our index patient represents the first use of AXL-targeted therapy in ACC, after confirmation of AXL-high IHC. While this is a single patient case, she encouragingly experienced pronounced tumor regression and marked symptom improvement, favorably compared with previous therapies. Even at PD after 12 months, she had lower disease burden and better quality of life than before AXL-directed therapy (Fig 4).

The dissociation between the significant clinical and radiologic improvement compared with the BOR of SD highlights important limitations of RECIST in assessing ACC polymetastatic pulmonary and pleural diseases, as use of only two TLs may lead to inaccurate assessments of response.^34^ In metastatic papillary thyroid cancer, which exhibits similar indolent and polymetastatic behavior, alternative methods of disease evaluation have been proposed, including tumor volume doubling time.^35-37^ Similar volumetric and metabolic-based response evaluations are being explored in ACC trials.^38,39^ In addition, considering the prolonged disease course of these patients, who live years with a cumulative increase in pulmonary tumor burden, quality-of-life and symptom management should be integrated into clinical decision making and as end points in clinical trials.

The AXL-targeted therapy used in this case was BA3011.^15,16,40^ The toxicity profile was favorable; she received just over a year of continuous therapy and came off trial for PD, not toxicity. While a causal association cannot be confirmed, the progressive increase in ADA during her last three cycles of treatment might have contributed to decreased ADC activity.^41^

In conclusion, to our knowledge, our patient is the first reported ACC case treated with AXL-targeted therapy. Her positive P63 IHC identified her ACC-II subtype, known to be AXL high. Our patient’s positive response and good tolerance to AXL-CAB-ADC, along with known AXL overexpression in most ACCs, support further investigation of AXL-targeted therapy for this orphan disease.

## Data Availability

The data sets used and/or analyzed during the current study are available from the corresponding author upon reasonable request.
